# A sarcopenia prediction model based on the calf maximum muscle circumference measured by ultrasound

**DOI:** 10.1186/s12877-025-05733-y

**Published:** 2025-02-05

**Authors:** An Wei, Yan Zou, Zhen-Hua Tang, Feng Guo, Yan Zhou

**Affiliations:** 1 Department of Ultrasound, Hunan Provincial People’s Hospital, The First Affiliated Hospital of Hunan Normal University, No.89, GuHan Avenue, Changsha, HuNan 410024 China; 2 Department of International Medicine, Hunan Provincial People’s Hospital, The First Affiliated Hospital of Hunan Normal University, Changsha, HuNan China; 3 Department of Geriatrics, Hunan Provincial People’s Hospital, The First Affiliated Hospital of Hunan Normal University, Changsha, HuNan China

**Keywords:** Sarcopenia, Prediction model, Calf muscle maximum circumference, Ultrasound, Hospitalized older patients

## Abstract

**Background:**

The correlation between calf circumference（CC）and sarcopenia has been demonstrated, but the correlation between calf maximum muscle circumference (CMMC) measured by ultrasound and sarcopenia has not been reported. We aims to construct a predictive model for sarcopenia based on CMMC in hospitalized older patients.

**Methods:**

This was a retrospective controlled study of patients > 60 years of age hospitalized in the geriatric department of Hunan Provincial People’s Hospital. The patients were thoroughly evaluated by questionnaires, laboratory, and ultrasound examinations, including measuring muscle thickness and calf muscle maximum circumference using ultrasound. Patients were categorized into sarcopenia and non-sarcopenia groups according to the consensus for diagnosis of sarcopenia recommended by the Asian Working Group on Sarcopenia 2019 (AWGS2). Independent predictors of sarcopenia were identified by univariate and multivariate logistic regression analyses, and a predictive model was developed and simplified. The prediction performance of the models was assessed using sensitivity, specificity, and area under the curve (AUC) and compared with independent predictors.

**Results:**

We found that patient age, albumin level (ALB), brachioradialis muscle thickness (BRMT), gastrocnemius lateral head muscle thickness (Glh MT), and calf maximum muscle circumference (CMMC) were independent predictors of sarcopenia in hospitalized older patients. The prediction model was established and simplified to Logistic *P* = -4.5 + 1.4 × age + 1.3 × ALB + 1.6 × BR MT + 3.7 × CMMC + 1.8 × Glh MT, and the best cut-off value of the model was 0.485. The sensitivity, specificity, and AUC of the model were 0.884 (0.807–0.962), 0.837 (0.762–0.911), and 0.927 (0.890–0.963), respectively. The kappa coefficient between this model and the diagnostic criteria recommended by AWGS2 was 0.709.

**Conclusion:**

We constructed a sarcopenia prediction model with five variables: age, ALB level, BR MT, Glh MT, and CMMC. The model could quickly predict sarcopenia in older hospitalized patients.

**Supplementary Information:**

The online version contains supplementary material available at 10.1186/s12877-025-05733-y.

## Introduction

Sarcopenia, first introduced by Irwin Rosenberg in 1989, is a progressive age-related systemic disorder characterized by a progressive loss of muscle mass, weight, and function, which is strongly associated with adverse outcomes such as falls, fractures, disability, and death [1, 2, 3].The global prevalence of sarcopenia has risen to 10-27% with the accelerated aging of the world population [4]. In China, the prevalence of sarcopenia in the community-based older adults population is approximately 17%, and the prevalence in long-term hospitalized patients could be as high as 40% [5, 6]. In addition, there are about 40 million disabled older adult in China who are unable to walk independently or hold correctly to perform the gait speed and grip strength measurement, so they cannot be diagnosed with sarcopenia. By 2050, the prevalence of sarcopenia is estimated to reach 500 million people [7].The physical decline and adverse events caused by sarcopenia have a significant economic impact on individuals, families, and society. The direct medical costs associated with sarcopenia were estimated by the US Department of Health and Social Care in 2000 to be approximately $18.5 billion [7]. Given that sarcopenia could be effectively treated with low-cost, non-invasive methods, early, proactive, and effective interventions become essential to avoid adverse outcomes of sarcopenia [8]. However, in many countries, sarcopenia is not routinely diagnosed due to the lack of practical clinical diagnostic tools [7].Computed tomography (CT), magnetic resonance imaging (MRI), and dual-energy X-ray absorptiometry (DXA) have been recommended by the European Working Group on Sarcopenia in the older adults (EWGSOP) as the gold standard for determining muscle mass. Still, the equipment is expensive, and the CT and X-rays are also radioactive, which is not conducive to routine screening. Measurement of muscle mass with Bioelectrical impedance analysis (BIA) may be superior to DXA but is also influenced by the hydration status of the patient [8, 9].

Portable ultrasounds are lightweight and easy to move to the community and other primary care units for screening. The latest version of the European Working Group on Sarcopenia in the older adults Consensus (EWGSOP2) recommends ultrasound as a valid and reliable tool for measuring muscle mass [10]. Researchers have chosen muscle thickness (MT), cross-sectional area (CSA), and echo intensity to assess muscle mass, but the optimal muscle group to reflect whole-body muscle mass has not been identified, and the diagnostic efficacy of the same parameter varies widely in different studies. In 2019, the Asian Working Group on Sarcopenia (AWGS2) suggested that the SARC-F scale and calf circumference (CC) should be combined to form the SARC-calF score or directly use CC to screen for sarcopenia [11]. CC includes subcutaneous fat, which is affected by the patient’s body fat rate, and it is easy to miss obesity and edematous sarcopenia [12]. Ultrasound can directly measure the circumference of the calf’s skeletal muscle without the interference of subcutaneous fat. We hypothesized that the calf muscle circumference could reflect the calf skeletal muscle mass more realistically because the interference of subcutaneous fat was excluded. Therefore, it could be more accurate in the diagnosis of sarcopenia. However, there are no reports on the prediction of sarcopenia by ultrasound-measured calf skeletal muscle circumference.

Our study aimed to construct a predictive model of sarcopenia by measuring the CMMC, BR MT, and lower limb MT by ultrasound, combined with the patient’s clinical basis, serum biochemistry, and lifestyle habits. We hypothesized that the predictive model incorporated into CMMC would have good predictive performance and high agreement with the diagnostic results of AWGS2. It can be applied to all older inpatients, especially those unable to perform gait speed or grip strength tests.

## Materials and methods

### Study subjects

This study was a controlled observational study conducted in accordance with the Declaration of Helsinki. It was reviewed and approved by the Medical Ethics Committee of Hunan Provincial People’s Hospital (approval number: LL-20250517-457). As this was not a prospective intervention study, no clinical study lot number was requested. All participants signed written informed consent.

Patients who were hospitalized in the Department of Geriatrics of our hospital from November 2021 to January 2023 and voluntarily underwent sarcopenia screening were selected. Inclusion criteria: (1) age ≥ 60 years old; (2) able to independently complete all motor function assessments required for sarcopenia screening; (3) be conscious and able to complete the questionnaire; Exclusion criteria: (1) Recent conditions that may cause drastic changes in body composition, such as acute systemic infection, electrolyte disturbance, massive blood loss, etc. (2) acute or chronic organ failure; (3) patients with stroke or hemiplegia cannot independently complete motor function assessment; (4) Unable to complete the questionnaire independently. After strict screening according to the inclusion and exclusion criteria, the AWGS (Asia Working Group for Sarcopenia) 2019 diagnostic consensus (AWGS2) was used as the “gold standard” [13], and all enrolled patients were divided into the sarcopenia group and the non-sarcopenia group.

### Equipment and methods

#### Clinical data collection

All patients were informed about the content and methods of the study and signed an informed consent form before participating in the study. The same physician measured the height and weight of the patients on the day of hospitalization, recorded their age and other basic clinical information, and drew venous blood from the patients early in the morning before the start of the treatment to test the biochemical indexes: albumin (ALB), triglyceride (TG), and so on.

#### Basic information questionnaire

A questionnaire survey was conducted in the awake state of the patients, and the intake of eggs and meat, and the number and duration of weekly exercises were recorded. The questionnaire was developed by us according to the research needs (shown in supplemental material 1).

#### HGS ( hand grip strength ) measurement

The patient was instructed to stand in a position with the shoulders retracted, the upper arms close to the median axillary line, the elbows flexed at 90 degrees, the long axis of the upper arms perpendicular to the floor, and the long axis of the forearms parallel to the floor. The data were read when the patient held the force surface of the grip strength device (RL-PG-07 from Guangzhou, China) with the dominant hand and tightened the handle device to the maximum extent. A total of 3 measurements were made and the maximum value was recorded.

#### Gait speed (GS) measurement

Measure the length of 6 m on flat, clean, and unobstructed ground and mark the starting point and the endpoint. Patients were asked to walk from the starting point to the endpoint at the normal speed of daily walking without slowing down in between, and the required time was recorded using a stopwatch for a total of two tests and averaged.

#### Skeletal muscle mass was measured by Bioimpedance analysis(BIA)

The skeletal muscle mass of the patients was measured using a Korean Jevon IO1 human tissue composition analyzer. During the examination, patients removed their shoes and socks after urination, took off their metal jewelry, stood calmly, and held the handle of the instrument with both hands to ensure that the skin on the soles of the feet and palms of the hands was in direct contact with the electrodes of the feet and the handle. The patient’s legs were slightly spread to ensure that the skin of the inner thighs did not come into direct contact with the electrodes, and the patient remained calm and did not move until the machine indicated that the measurement had been completed, obtaining the patient’s body weight, fat mass, defatted mass, and muscle mass of the limbs.

Appendicular skeletal muscle mass (ASM) was calculated as the sum of the appendicular skeletal muscle muscle mass (kg) measured by BIA divided by the square of the height (m) (kg /m²).

#### Ultrasonic measurement

Mindray M9 ultrasound instrument (Myriad Medical International Ltd, Shen Zhen, China) was used to measure the muscle thickness (MT) of bilateral brachioradialis (BR), vastus medialis (VM), gastrocnemius medial head (Gmh) and gastrocnemius lateral head (Glh) in the resting state of patients with L12-4 high-frequency superficial probe.

#### Muscle measurement methods

**Fig. 1:**
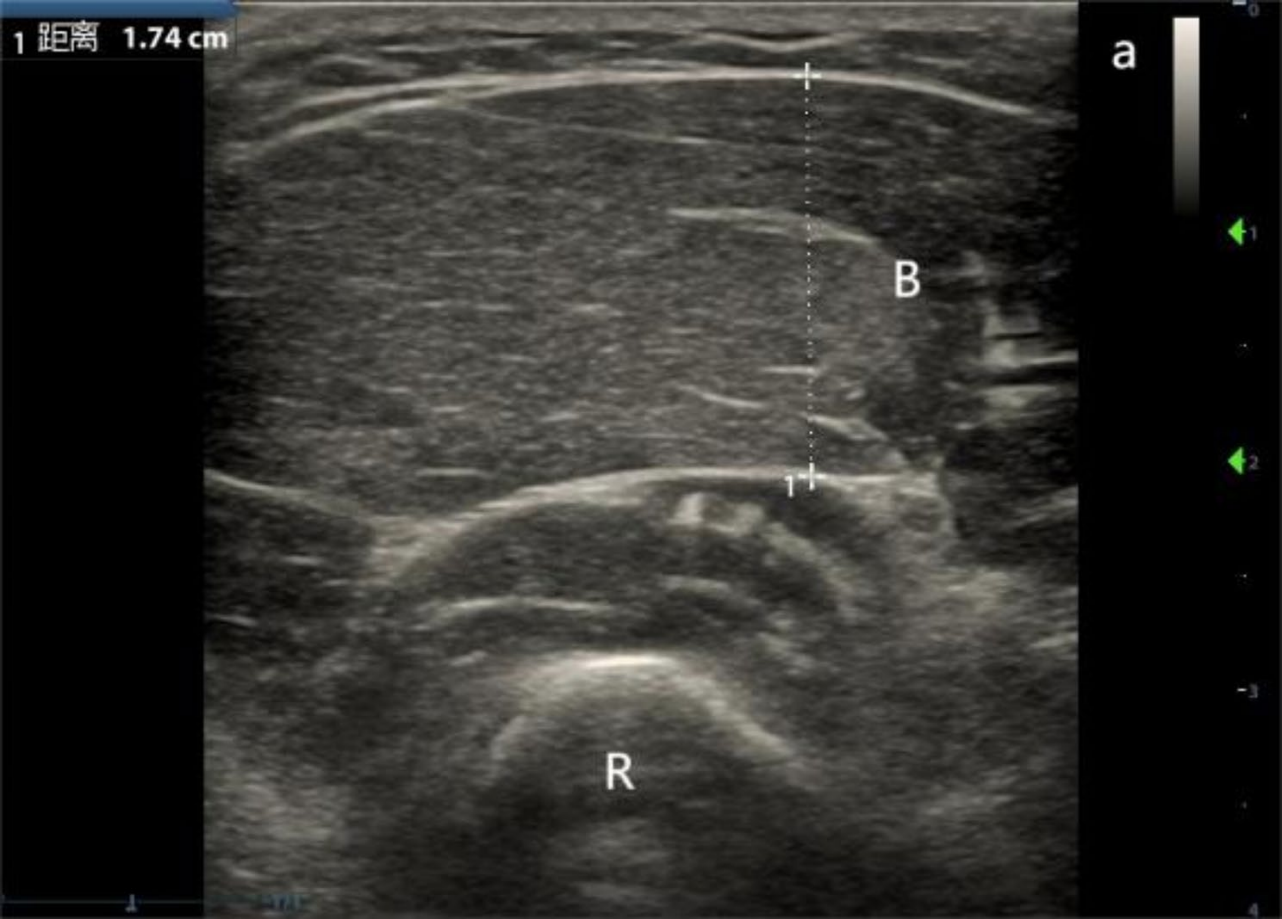
Measurement of BR MT (B: brachioradialis muscle, R: radius)

**Fig. 2:**
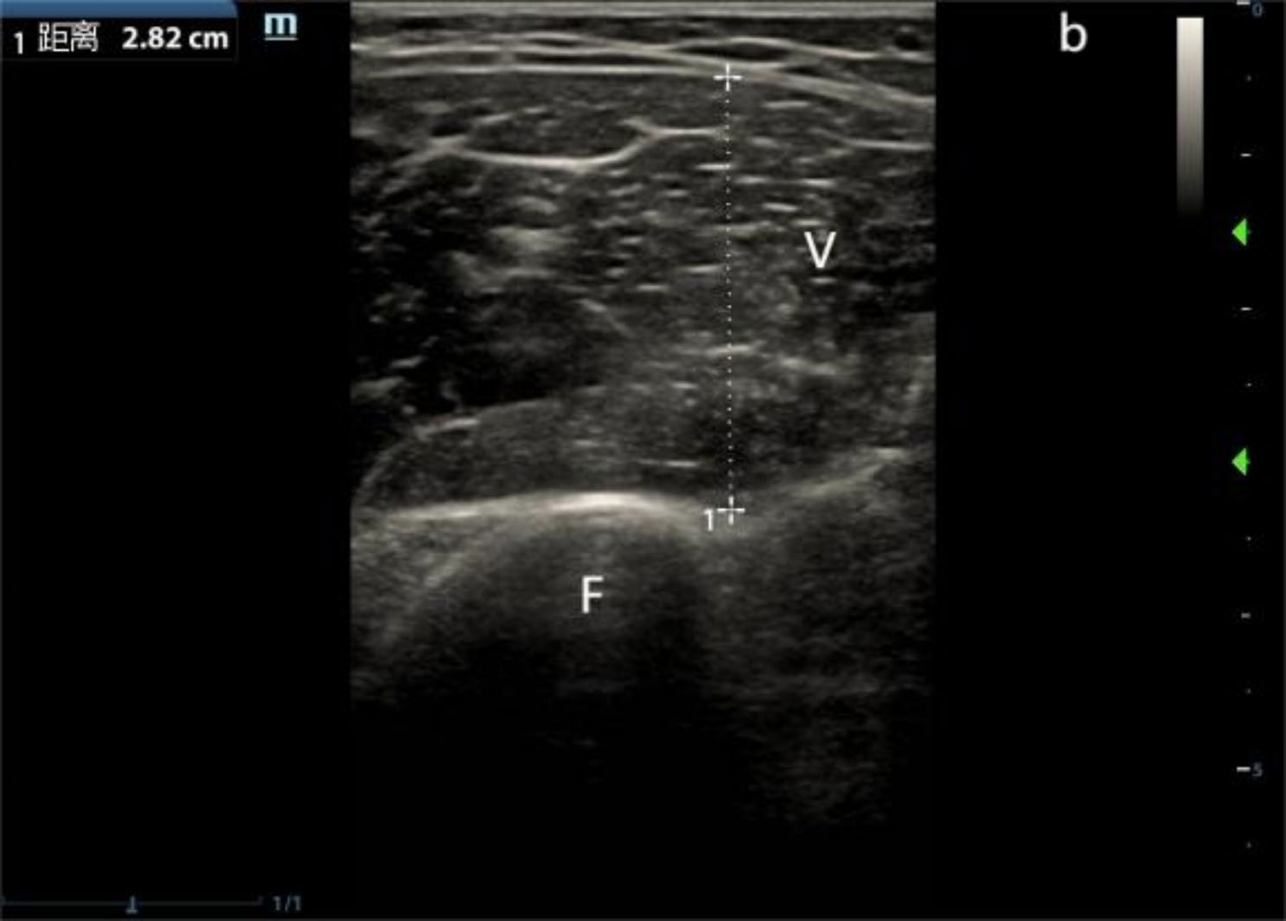
Measurement of VM MT (V: Vastus medialis muscle, F:Femur)

**Fig. 3:**
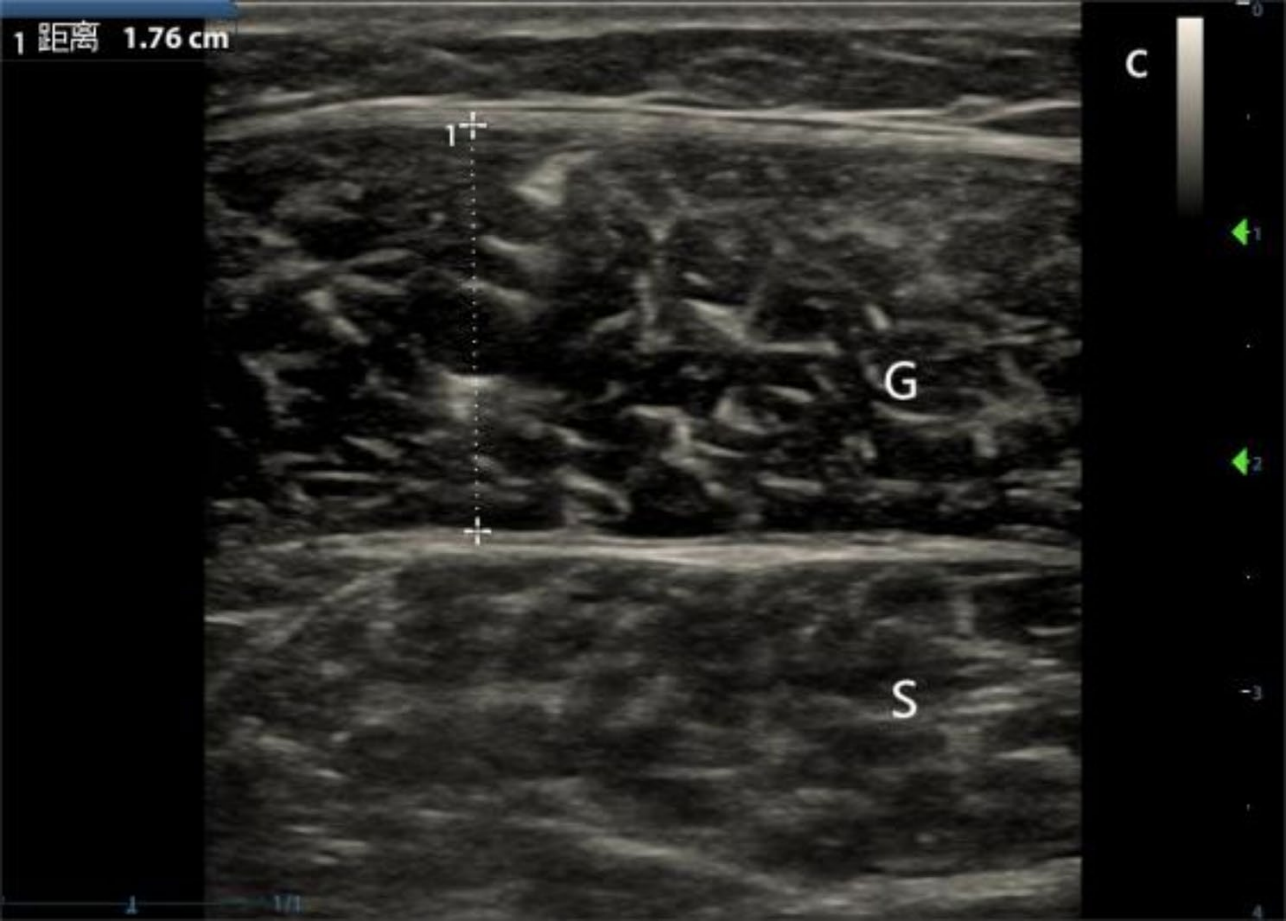
Measurement of Gmh MT (G: gastrocnemius medial head, S: Soleus )

**Fig. 4:**
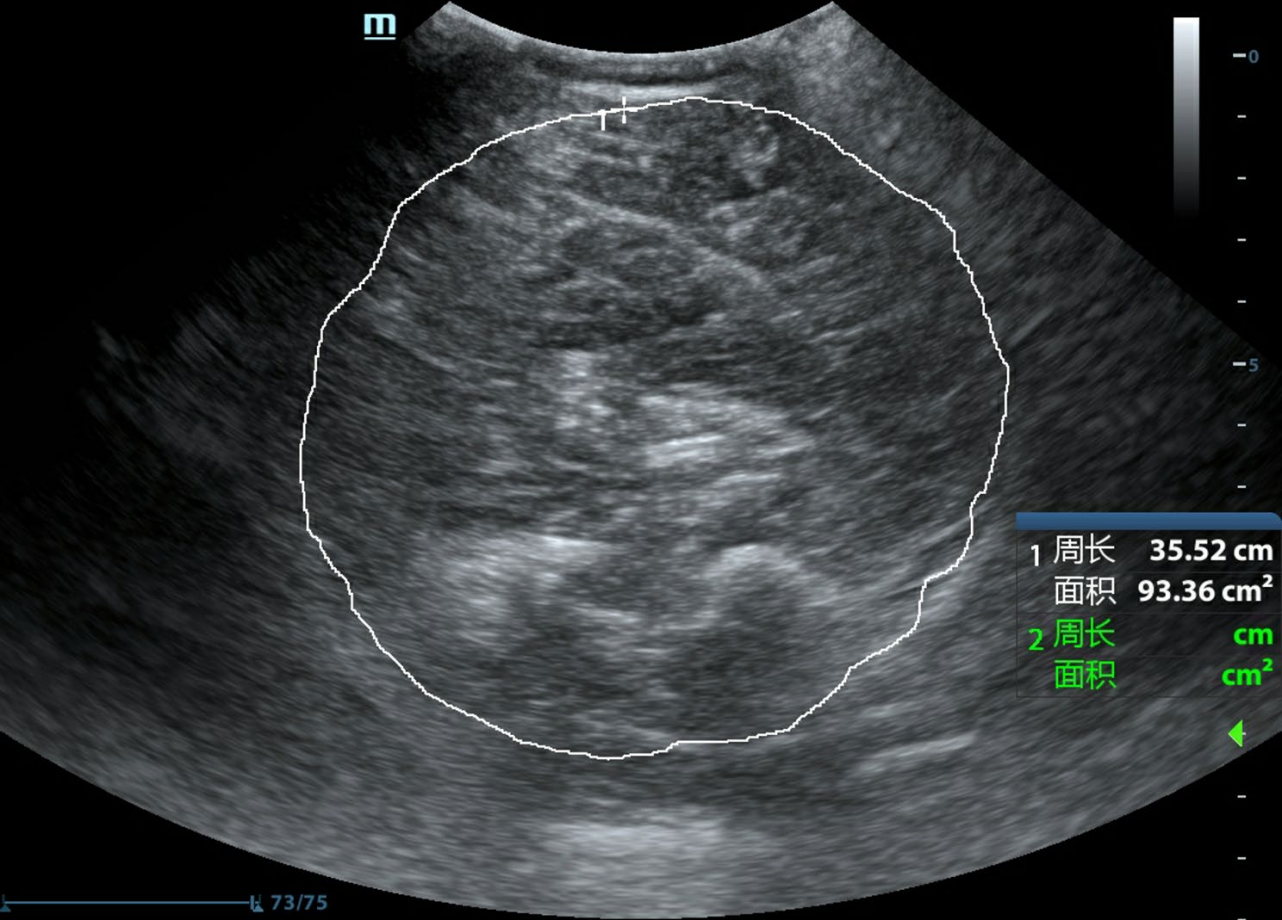
Measurement of CMMC. The white line was the trajectory depicted when the maximum circumference was measured around the periphery of the calf muscle using the trajectory method

1. Brachioradialis (BR): The patient was placed in the supine position with the hands flat on either side of the body, palms naturally facing upward, and the probe was placed in the proximal 1/3 of the radius between the radial head and the condylar eminence of the carpal tunnel, with the long axis of the probe perpendicular to the long axis of the forearm. The MT of the BR was measured by scanning over an area of 6 centimeters above and below the BR, taking the largest cross-section of the BR (as show in Fig. 1).

2. Vastus medialis (VM): the patient lies supine with the knee bent at 120° and the legs slightly separated. The probe was placed horizontally at the distal 1/3 of the inner thigh, and the scanning range was 6 cm up and down. The MT of the VM was measured at the maximum cross-section (as show in Fig. 2).

3. Gastrocnemius medial head (Gmh) and Gastrocnemius lateral head (Glh): the patient lies in the supine position with both knees bent at 120 degrees and legs slightly separated by 30 degrees—probe on the lateral and medial crus, popliteal fossa proximal 1/3, and tibial articular surface level. The longitudinal axis of the probe was perpendicular to the long axis of the calf. The scan range was 6 cm above and below, and MT was measured at the thickest point of the Glh and Gmh (as show in Fig. 3).

4. Calf muscle maximum circumference (CMMC): A C5-1 abdominal probe was selected and extended to the maximum imaging angle, and the probe was perpendicular to the calfskin. The cross-section with the largest display of the calf muscle group was selected, and the boundary between the muscle and the subcutaneous fat was clear. Then the measurement cursor was traced around the lateral edge of the calf muscle group by manual tracing, and the CMMC was obtained (as shown in Fig. 4).

#### Quality control

(1) All instruments are calibrated before use to ensure optimal functioning. (2) questionnaires were conducted and recorded by the same trained professional to ensure consistency. (3) all ultrasound images are captured and stored by a physician trained in musculoskeletal ultrasound with at least one year of experience in diagnostic ultrasound to ensure standardization of sections and clear imaging. (4) During the ultrasound procedure, a thick layer of coupling agent is applied between the probe and the skin to prevent external forces from interfering with the imaging. (5) Ultrasound data are averaged by the same sonographer after three repeated measurements on the stored images, retaining one decimal place.

#### Diagnostic criteria

The consensus for the diagnosis of Sarcopeniarecommended by the Asia Working Group for Sarcopenia in 2019 ( AWGS2) was used as the “gold standard” for this study. For details, see the figure:

(1) ASM ≤ 7.0 kg/m^2^ in men and ≤ 5.7 kg/m^2^ in women (measured by BIA);

(2) HGS < 28 kg in males and < 18 kg in females;

(3) GS < 1.0 m/s;

If it was 1 + 2/3, it could be judged as sarcopenia, and all patients were divided into the sarcopenia group and the non-sarcopenia group.

### Statistical methods

SPSS 26.0 software was utilized for conducting statistical analysis. The Shapiro-Wilk test was employed to assess the normality of the data distribution. In cases where the data followed a normal distribution, intergroup comparisons were performed using an independent t-test. For non-normally distributed data, non-parametric tests were applied for comparison purposes. Frequency was used to express count data, and group comparisons were analyzed using the chi-square test. Univariate and multivariate logistic regression analyses were conducted to identify independent predictors of sarcopenia in hospitalized older adults patients, leading to the development of a predictive model. The predictive performance of individual predictors and the model was evaluated through receiver operating characteristic (ROC) curves analysis. Statistical significance was defined as *P* < 0.05.

At present, the 10EPV (10eventspervariable) method is the most widely used empirical rule for sample size calculation in the development of clinical prediction models. It is defined as ensuring that each predictor variable in the final regression equation corresponds to at least 10 outcome events. According to previous literature, the incidence of sarcopenia in hospitalized elderly patients is 40%. We expected the prevalence of sarcopenia to be 35%. The final variables that entered the multivariate logistic regression model were five or fewer. To allow the entry of five predictors in the final model, based on the 10EPV method and an outcome event rate of 35%, we estimated that at least 143 participants would need to be enrolled.

## Results

**Fig. 5:**
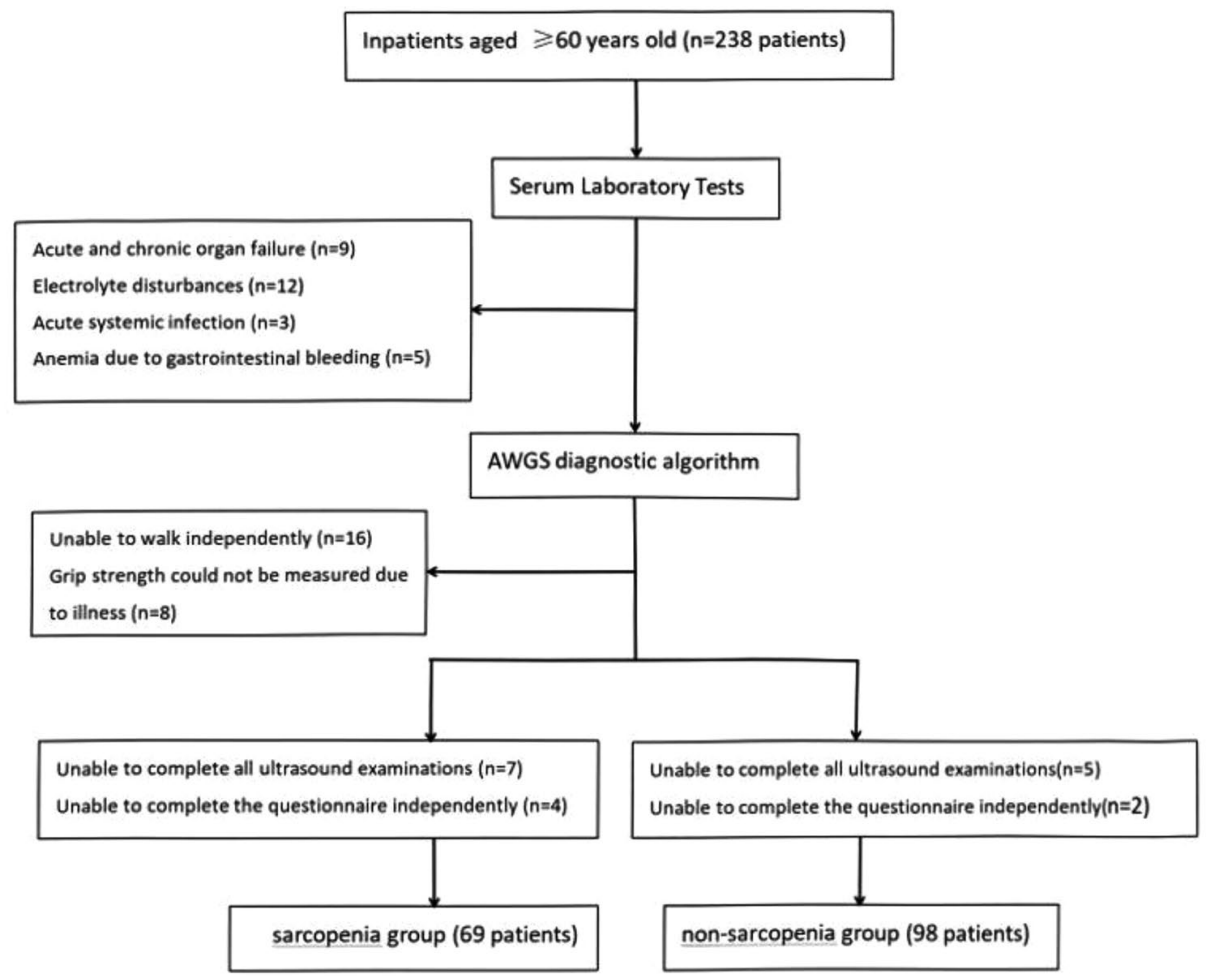
Patient selecion flow chart of this study

Finally, a total of 167 patients were included in this study (shown in Fig. 5). The sample size exceeded the estimated amount of the 10EPV method, so the estimation accuracy of the key parameters in the prediction model was guaranteed.

The age of 167 patients was (70.9 ± 8.1) years, including 91 males (70.9 ± 8.2) years and 76 females (70.9 ± 7.9) years. Using AWGS2 as the “gold standard”, 69 patients with sarcopenia and 98 patients without sarcopenia were diagnosed. The prevalence of sarcopenia was 41.3% (male: 49.5%, female: 31.5%), and there was a gender difference in the incidence of sarcopenia (*P* = 0.020).

### Comparison of clinical basis, serum biochemical between the Sarcopenia and non-sarcopenia groups

The comparison of clinical basis and serum biochemical between the sarcopenia and non-sarcopenia groups is shown in Tables 1 and 2. There were significant differences in gender, age, and ALB between the two groups. The incidence of sarcopenia in men was 49.5%, which was significantly higher than that in women (31.5%, *P* = 0.020). The average age of the sarcopenia group was also higher than that of the non-sarcopenia group (73.4 vs.69.1, *P* = 0.002). The average ALB level in the sarcopenia group was 39.14 g/L, lower than 41.77 g/L in the non-sarcopenia group (*P* < 0.001). The continuous variables were converted into binary variables by drawing the ROC curve to obtain the best cut-off value for each variable. The optimal cut-off values of age and ALB were 70 years and 40 g/L, respectively.

**Table 1:** Comparison of clinical basis, serological biochemical and ultrasound measurements

variables			Sarcopenia (*n* = 69)		Non-sarcopenia (*n* = 98)		Z/X^2^	*P* value
Gender	male(*n* = 91)		45(49.5%)		46(50.5%)		5.4555	0.020
	female(*n* = 76)		24(31.5%)		53(68.4%)			
Age(year)			73.4 ± 8.7		69.1 ± 7.2		-3.106	0.002
Weight(kg)			62.26 ± 11.03		64.60 ± 10.22		-0.761	0.447
Height(cm)			162.7 ± 6.8		162.9 ± 8.8		-0.111	0.912
BMI			23.34 ± 2.46		24.24 ± 2.68		-1.745	0.081
GS(m/s)			0.75 ± 0.20		0.89 ± 0.15		-5.044	<0.001
		M	0.75 ± 0.19	0.922^*^	0.89 ± 0.14	0.475^*^	-3.467	0.001
		F	0.75 ± 0.21		0.90 ± 0.15		-3.209	0.001
HGS(Kg)			22.13 ± 6.72		23.86 ± 5.66		-1.790	0.075
		M	25.36 ± 5.74	<0.001^*^	28.56 ± 3.65	<0.001^*^	-3.164	0.002
		F	16.08 ± 3.42		19.69 ± 3.40		-4.035	<0.001
ASM(kg/m²)			6.10 ± 0.78		7.16 ± 0.86		-6.756	<0.001
		M	6.54 ± 0.45	<0.001^*^	7.83 ± 0.50	<0.001^*^	-8.217	<0.001
		F	5.28 ± 0.57		6.57 ± 0.65		-6.683	<0.001
Glu(mmol/L)			6.68 ± 2.83		7.04 ± 3.69		-0.34	0.734
TG (mmol/L)			1.63 ± 1.03		1.77 ± 1.22		-0.816	0.415
VLDL(mmol/L)			0.83 ± 0.35		0.80 ± 0.30		-0.124	0.902
LDL(mmol/L)			2.71 ± 0.72		2.70 ± 1.02		-0.874	0.382
HDL(mmol/L)			1.23 ± 0.35		1.17 ± 0.29		-0.478	0.633
TC(mmol/L)			4.68 ± 0.98		4.59 ± 1.29		-0.926	0.354
GLB(g/L)			27.22 ± 3.63		27.22 ± 3.61		-0.059	0.953
ALB(g/L)			39.14 ± 2.85		41.77 ± 2.85		-4.954	<0.001
TP(g/L)			68.32 ± 5.13		67.52 ± 9.12		-0.221	0.825
CMMC(cm)			24.93 ± 3.74		28.4 ± 2.66		-5.613	<0.001
		M	24.97 ± 3.70	0.901^*^	28.28 ± 3.06	0.631^*^	-4.641	<0.001
		F	24.85 ± 3.89		28.54 ± 2.28		-4.311	<0.001
Glh MT(mm)			13.45 ± 2.55		15.86 ± 2.07		-6.083	<0.001
		M	13.31 ± 2.47	0.531^*^	15.65 ± 1.90	0.350^*^	-5.062	<0.001
		F	13.72 ± 2.72		16.04 ± 2.21		-3.955	<0.001
Gmh MT(mm)			13.42 ± 2.55		15.84 ± 2.08		-6.092	<0.001
		M	13.28 ± 2.47	0.525^*^	15.63 ± 1.91	0.353^*^	-5.082	<0.001
		F	13.69 ± 2.73		16.02 ± 2.22		-3.951	<0.001
VM MT(mm)			25.29 ± 6.00		25.14 ± 5.10		-0.137	0.891
		M	25.51 ± 5.92	0.974^*^	25.11 ± 5.07	0.953^*^	0.173	0.863
		F	25.25 ± 6.27		25.17 ± 5.17		0.064	0.949
BR MT(mm)			13.67 ± 2.80		15.42 ± 2.20		-3.972	<0.001
		M	14.08 ± 2.84	0.095^*^	15.37 ± 2.13	0.837^*^	-2.459	0.016
		F	12.90 ± 2.62		15.46 ± 2.27		-4.356	<0.001
Meat intake per meal	Y *n* = 71		19(27.5%)		52(72.5%)		10.794	0.001
	N *n* = 96		50(53.1%)		46(46.9%)			
Weekly exercise duration<90 min	Y *n* = 43		27(62.8%)		16(37.2%)		1.013	0.001
	N *n* = 124		42(33.9%)		82(66.1%)			
Weekly exercise ≥ 3 times	Y *n* = 123		47(38.2%)		76(61.8%)		1.857	0.173
	N *n* = 44		22(50.0%)		22(50.0%)			
Calcium supplements ≥ 600 mg	Y *n* = 33		13(39.4%)		20(60.6%)		0.063	0.082
	N *n* = 134		56(41.8%)		78(58.2%)			
One egg intake per day	Y *n* = 100		42(42.0%)		58(58.0%)		0.048	0.827
	N *n* = 67		27(40.3%)		40(59.3%)			

BMI: Body mass index Glu: glucose TG: Triglyceride VLDL: very low density lipoprotein

LDL: low density lipoprotein HDL: high density lipoprotein TC total cholesterol GLB: globulin

ALB: albumin TP: total protein CMMC: Calf muscle maximum circumference MT: muscle thickness

Glh: gastrocnemius lateral head Gmh: gastrocnemius medial head VM: vastus medialis BR: brachioradialis

GS: Gait speed HGS: Hand grip strength ASM: Appendicular skeletal muscle mass * *P* values for comparisons between sexes

**Table 2:** Best cut-off values of each variable

Variable	Youden index	Best cut-off value	Layer value (1=,else = 0)	Sensitivity ( % )	Specificity (% )	AUC
Age(year)	0.218	69.5	>70	66.7	55.1	0.641 ( 0.557–0.725 )
ALB(g/L)	0.463	39.8	<40	79.6	66.7	0.725 ( 0.644–0.806 )
Gmh MT(mm)	0.436	15.2	<15	65.3	78.3	0.777 ( 0.705–0.849 )
Glh MT(mm)	0.436	15.2	<15	65.3	78.3	0.777 ( 0.705–0.849 )
BR MT(mm)	0.401	15.2	<15	63.3	76.8	0.681 ( 0.594–0.768 )
CMMC(cm)	0.524	24.3	<24	95.9	56.5	0.755 ( 0.675–0.836 )

ALB: albumin MT: muscle thickness Gmh:gastrocnemius medial head Glh: gastrocnemius lateral head BR: brachioradialis CMMC: Calf muscle maximum circumference

### Comparison of ultrasound measurements between the Sarcopenia and non-sarcopenia groups

The comparison of ultrasound measurements between the sarcopenia and non-sarcopenia groups is shown in Tables 1 and 2. There were significant differences in CMMC, BR MT, Glh MT, and Gmh MT between the two groups, but no significant differences in the remaining variables. The BR MT, Glh MT, Gmh MT, and CMMC of the sarcopenia group were lower than those of the non-sarcopenia group. The best cut-off values of BR MT, Gmh MT, and Glh MT were all 15 mm, and the best cut-off value of CMMC was 24 cm. CMMC < 24 cm had the highest positive predictive value (90.7%), sensitivity (95.9%), and negative predictive value (75.8%) for sarcopenia. Its Kappa value with the gold standard diagnosis was 0.555, better than other skeletal muscle variables.

As shown in Table 1, there were no significant differences in BR, VM, Gmh, Glh, and CMMC values between males and females in the sarcopenia group and the non-sarcopenia group, *P* values were 0.095, 0.974, 0.525, 0.531, 0.901 and 0.837, 0.953, 0.353, 0.350, 0.631, respectively.

### Comparison of lifestyle habits between Sarcopenia and non-sarcopenia groups

The comparison of lifestyle habits between the sarcopenia and non-sarcopenia groups is shown in Tables 1 and 3. There were differences in meat intake per meal and weekly exercise time between the two groups. The proportion of meat intake per meal and weekly exercise duration in the non-sarcopenia group was higher than in the sarcopenia group. No meat intake per meal was associated with a 1.8-fold increased risk of sarcopenia. Those who exercised ≤ 90 min weekly had about a 1.3-fold increased risk for sarcopenia. There were no significant differences in the number of weekly exercises, calcium supplementation, and one egg intake per day between the two groups.

**Table 3:** Binary logistic regression analysis of statistically significant variables

Variable		Univariate analysis			Multifactor analysis		
		*P* value	OR	95% CI	*P* value	OR	95% CI
Gender		0.020	2.120	1.124–3.998	0.354	1.585	0.598 ~ 4.197
Age > 70 years old		0.006	2.455	1.295 ~ 4.653	0.008	3.949	1.430 ~ 10.902
ALB<40 g/L		<0.001	7.048	3.538 ~ 14.039	0.008	3.637	1.392 ~ 9.505
BRMT<15 mm		<0.001	4.021	2.088 ~ 7.744	0.002	4.818	1.761 ~ 13.187
CMMC<24 cm		<0.001	30.550	10.088 ~ 92.519	<0.001	38.659	10.135 ~ 147.457
Glh MT<15 mm		<0.001	5.844	2.951 ~ 11.574	0.001	6.102	2.100 ~ 17.731
Gmh MT<15 mm		<0.001	5.758	2.895 ~ 11.452	0.979	1.061	0.013 ~ 88.396
Not eating meat at every meal		0.001	2.975	1.537 ~ 5.759	0.264	1.790	0.644 ~ 4.973
Weekly exercise duration<90 min		0.001	3.295	1.601 ~ 6.779	0.571	1.343	0.484 ~ 3.726
Constant quantity	-4.449				<0.001	0.012	

VLDL: very low density lipoprotein LDL: low density lipoprotein HDL: high density lipoprotein TC: total cholesterol

GLB: globulin ALB: albumin TP: total protein CMMC: Calf muscle maximum circumference MT: muscle thickness

Glh: gastrocnemius lateral head Gmh: gastrocnemius medial head VM: vastus medialis BR: brachioradialis

### Construction of sarcopenia prediction models

The statistically different dichotomous variables were included in the multivariate binary logistic regression analysis, and the results are shown in Table 3. Finally, age, ALB level, BR MT, Glh MT, and CMMC were independent predictors of sarcopenia. The prediction model was constructed as follows: Logistic *P* = − 4.449 + 1.373×Age + 1.291× ALB + 1.572× BR MT + 3.655× CMMC + 1.809 × Glh MT. When the Youden index was 0.721, the best cut-off value of the model was 0.485, the prediction sensitivity was 0.884 (0.807–0.962) and the specificity was 0.837 (0.762–0.911). The positive predictive value was 79.2%, the negative predictive value was 91.1%, and the Kappa value was 0.709 (0.654–0.764). For the convenience of clinical use, the model was simplified as follows: Logistic *P* = − 4.5 + 1.4 × Age + 1.3× ALB + 1.6 × BR MT + 3.7×CMMC + 1.8×Glh MT. The cut-off value of the simplified model was 0.485, and the sensitivity and specificity were 0.884 and 0.837, respectively. Compared with the original model, the values were unchanged.

### ROC analysis of the independent predictors and the models

**Fig. 6:**
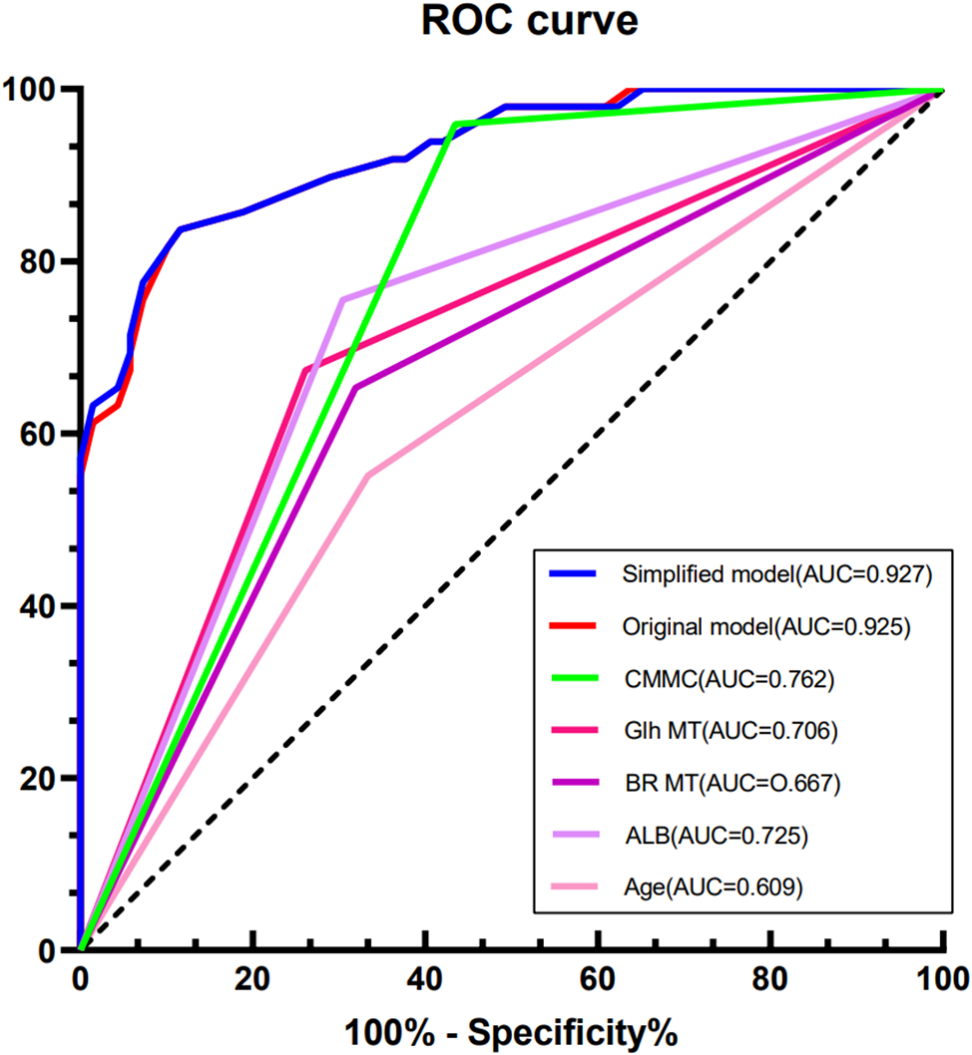
The AUC of the model was larger than that of all single predictors

The ROC analysis of single independent predictors of sarcopenia and the models are shown in Table 4; Fig. 6. The area under the ROC curve (AUC) of the simplified model and the original model was larger than that of the single predictor, and the simplified model was slightly higher than the original model ( AUC = 0.927 vs.0.925 ).

**Table 4:** ROC analysis of individual predictors and models

Variable	AUC	*P* value	95% CI	
			Lower limit	upper limit
simplified model	0.927	<0.001	0.890	0.963
original model	0.925	<0.001	0.888	0.962
Age	0.609	0.017	0.522	0.695
Glh MT	0.706	<0.001	0.626	0.787
CMMC	0.762	<0.001	0.683	0.842
BR MT	0.667	<0.001	0.583	0.751
ALB	0.725	<0.001	0.645	0.805

Note: the independent variables in this table are binary variables based on the optimal truncation value

ALB: albumin CMMC: Calf muscle maximum circumference MT: muscle thickness Glh: gastrocnemius lateral head

BR: brachioradialis

## Discussion

With the aging of the population, the incidence of sarcopenia is increasing year by year, especially in the hospitalized population, the incidence can be as high as 40% [6]. According to AWGS2, the diagnosis of sarcopenia has certain requirements for the examiner and is not suitable for patients with limited mobility [13]. To predict sarcopenia in older inpatients with limited mobility, we developed a sarcopenia prediction model based on data from serological examination and ultrasound, which had good sensitivity (0.884) and specificity (0.837). The kappa coefficient between this model and the diagnostic criteria recommended by AWGS2 was 0.709.

For the assessment of skeletal muscle mass, AWGS2 recommended DXA and BIA in 2019, but the measurement values of different brands are inconsistent [8, 14]. CC is the most convenient method for screening sarcopenia in primary medical institutions and communities. However, CC contains subcutaneous fat, which is affected by the body fat rate of patients, and it is easy to miss the diagnosis of obese sarcopenia [10, 13, 15]. Because ultrasound is portable, radiation-free, and can be performed at the bedside, the 2019 edition of EWGSOP2 recommends the use of ultrasound to assess muscle status [8, 16]. In Patrick Casey’s meta-analysis, the intraclass correlation coefficients of ultrasound measurements in multiple studies ranged from 0.82 to 0.99, indicating good reliability and reproducibility [17].

At present, there is no consensus on the best muscle, parameters, and thresholds for predicting sarcopenia, and most data are from Western populations. There are few studies on the Asian population, and AWGS2 recommends that more research data be needed to support the assessment of skeletal muscle mass by ultrasound. The ultrasound measurements associated with sarcopenia included MT, cross-sectional area(CSA), echo intensity, fascicle length, and pennation angle. However, the most studied are MT and CSA. Ryo Sato, a Japanese scholar, used a skin-measuring instrument to measure subcutaneous fat thickness. Then the CMMC was calculated according to the formula after subtracting the subcutaneous fat thickness from CC, which confirmed that the Kappa value between CMMC and AWGS2 diagnostic criteria for sarcopenia was 0.80 [6, 18]. By reviewing the previous studies, we selected BR MT, VM MT, Ghl MT, Gml MT, and CMMC which were associated with HGS, GS, and CC, respectively [19, 20, 21, 22].Our finding that BR MT is an independent predictor of sarcopenia is consistent with previous findings on the association of forearm muscle groups with grip strength [23]. In addition, CMMC, Glh MT, and Gmh MT are also independent predictors for sarcopenia in older adults. In our study, when Ghl MT, Ghl MT, and CMMC were lower than the optimal cut-off values, the risk of sarcopenia increased by 6 times, 4.8 times, and 38 times, respectively. CMMC had the best PPV and sensitivity and was moderately consistent with the diagnostic gold standard.

Quadriceps femoris is a muscle that has received more attention in previous studies, and about 70% of the studies have confirmed that it is associated with sarcopenia [17]. However, most of the studies included are Western populations, and there is a relative lack of data on Asian populations, and the data of its model is limited [9, 17]. A meta-analysis comparing 17 studies also found that the calf muscle group had higher diagnostic accuracy for sarcopenia than the quadriceps muscle group [10]. CMMC and Ghl MT were more strongly associated with sarcopenia than VMMT. This may be because older adults in China do not attach much importance to strength training, such as training the quadriceps femoris to stabilize the knee joint.

Previous studies have found that gender affects the occurrence of sarcopenia [24]. However, gender was not included in the multivariate analysis in our study. As can be seen from Table 1, there is no significant difference in the gender distribution of all skeletal muscle indexes, which is related to the national conditions of our country and the living habits of men and women. For example, women undertake more housework and are the main force of square dancing, resulting in women’s daily exercise volume being higher than men’s. However, selection bias and the small number of women may also impact the results, and further analysis with larger samples is needed.

The risk of sarcopenia is increased fourfold in people older than 70 years of age, suggesting that the rate of skeletal muscle loss increases after 70 years of age. However, when ALB < 40 g/L (normal range: 35 to 50 g/L), the risk of sarcopenia increases by 3.6 times, which is consistent with the report of Japanese scholars [25, 26]. However, the risk of sarcopenia increased by 1.8 times when meat was not consumed at each meal. Therefore, the older adults need to consume enough high-quality protein to ensure that ALB is maintained at a high level, especially meat [27, 28]. However, whether meat intake is more beneficial to the prevention of sarcopenia needs to be further verified.

In addition to diet, exercise can also effectively prevent the loss of muscle mass [29, 30, 31, 32]. We found that those who exercised ≤ 90 min per week had an approximately 1.3-fold increased risk of sarcopenia. Therefore, older adults, especially those over 70 years old, only need to exercise for up to 90 min per week to better prevent skeletal muscle loss.

Finally, our reduced model, which included five variables: age, ALB, BF MT, GlhMT, and CMMC, showed good predictive performance. The AUC was 0.927, the sensitivity was 88.4%, and the specificity was significantly higher than that of previous studies (88.4% vs. 50%, 68.1%) [33, 34]. The results showed a high consistency with the AWGS2 gold standard.

There are some limitations to our study. First, the data came from a small sample in one hospital, and it remains to be verified whether they apply to different populations in other healthcare organizations; second, there are additional factors that may affect sarcopenia that were not considered, such as smoking and alcohol consumption. Third, this study required patients to complete a physical fitness test and questionnaire, which was relatively time-consuming, and some patients were excluded because they did not complete all of them for a variety of reasons, so there was a case selection bias. Finally, external validation of the model validity has not yet been performed, which will be done in a follow-up study.

## Conclusion

In conclusion, we constructed a sarcopenia prediction model based on ALB, MT, and CMMC, which has good predictive performance and provides a relatively simple sarcopenia prediction tool for older patients, especially older inpatients. In addition, we found that eating meat with each meal and exercising for more than 90 min per week could reduce the risk of sarcopenia, which has a certain guiding significance for older hospitalized patients.

## Electronic supplementary material

Below is the link to the electronic supplementary material.

Supplementary Material 1

Supplementary Material 2

## Data Availability

Data is provided within the supplementary information files.

## Abbreviations

ALB: Albumin
ASM: Appendicular Skeletal Mass
AWGS: Asia Working Group for Sarcopenia
BIA: Bioimpedance analysis
BMI: Body Mass Index
BR: Brachioradialis
CI: Confidence interval
CC: Calf circumference
CMMC: Calf muscle maximum circumference
CSA: Cross-sectional area
CT: Computed tomography
DXA: Dual-energy X-ray absorptiometry
EWGSOP: the European Working Group on Sarcopenia in the older adults
FMI: Fat-Free Mass Index
GLB: Globulin
GS: Gait speed
Glh: Gastrocnemius lateral head
Glu: Glucose
Gmh: Gastrocnemius medial head
HDL: High density lipoprotein
HGS: Hand grip strength
LDL: Low density lipoprotein
MRI: Magnetic resonance imaging
MT: Muscle thickness
ROC: Receiver operating characteristic
SMI: Skeletal Muscle Mass Index
TC: Total cholesterol
TG: Triglyceride
TP: Total protein
VLDL: Very low density lipoprotein
VM: Vastus medialis
