# Supplementary material for: A sarcopenia prediction model based on the calf maximum muscle circumference measured by ultrasound
Source: BMC Geriatr. 2025 Feb 5;25:81. doi: 10.1186/s12877-025-05733-y (PMC11796220; doi:10.1186/s12877-025-05733-y)
Supplement: Supplementary file 1 — Supplementary Material 1 [file 12877_2025_5733_MOESM1_ESM.doc]

**Sarcopenia Status questionnaire**

Dear Sir/madam:

Hello! This survey is designed to better understand the incidence of sarcopenia in the elderly hospitalized, strengthen the awareness of the elderly on the prevention of sarcopenia, so as to improve the quality of life of the elderly. We need your cooperation to complete the relevant questionnaire and examination. Questionnaire will be strictly confidential, only used in scientific research, will not cause any adverse effects to you, please fill it out objective and true. Your participation will provide effective help for us to formulate the prediction, prevention and intervention of sarcopenia in the elderly. Thank you for your cooperation and support!

The survey need 10-15 minutes!

Are you willing to accept the questionnaire( Please check the alternative answer):

**Yes**( ) **no** ( )

signature：

Name:

Gender: Female( ) male( )

Age:

Height: cm

Body weight: Kg

BMI:

Ultrasonic measurement value:

Brachioradialis thickness： mm

Vastus medialis thickness： mm

Gastrocnemius medial head thickness： mm

Gastrocnemius lateral head thickness： mm

Calf muscle maximum circumference： cm

A, eating habits

1, daily protein intake:

Egg intake: ≥1 egg per day  yes( ), no( )

How many eggs intake per week on average？ （ ）

2, Meat intake per meal: yes( ), no( )

1. Calcium supplement: fill in the name of the drug ( )

How many pieces/pills per day ( )

B, Erexercise habits

1. The average number of times per week：

1. Each exercise duration: min

C, Serum biochemical records (completed by the investigator)

Total protein(TP): g/L

Albumin (ALB): g/L

Globulin (GLB): g/L

Total cholesterol (TC)： mmol/L

High density lipoprotein(HDL)： mmol/L

Low density lipoprotein(LDL)： mmol/L

Very low density lipoprotein(VLDL)： mmol/L

Triglycerides (TG)： mmol/L

Thank you for your cooperation and support again!

Time: date: ( ) month: ( )year: ( )

Signature of Investigator:
